# Noise-resilient and high-speed deep learning with coherent silicon photonics

**DOI:** 10.1038/s41467-022-33259-z

**Published:** 2022-09-23

**Authors:** G. Mourgias-Alexandris, M. Moralis-Pegios, A. Tsakyridis, S. Simos, G. Dabos, A. Totovic, N. Passalis, M. Kirtas, T. Rutirawut, F. Y. Gardes, A. Tefas, N. Pleros

**Affiliations:** 1 Department of Informatics, Aristotle University of Thessaloniki, 54124 Thessaloniki, Greece; 2 Center for Interdisciplinary Research and Innovation, Aristotle University of Thessaloniki, Thessaloniki, Greece; 3 Optoelectronics Research Centre, University of Southampton, Southampton, SO17 1BJ UK

**Keywords:** Applied optics, Computer science

## Abstract

The explosive growth of deep learning applications has triggered a new era in computing hardware, targeting the efficient deployment of multiply-and-accumulate operations. In this realm, integrated photonics have come to the foreground as a promising energy efficient deep learning technology platform for enabling ultra-high compute rates. However, despite integrated photonic neural network layouts have already penetrated successfully the deep learning era, their compute rate and noise-related characteristics are still far beyond their promise for high-speed photonic engines. Herein, we demonstrate experimentally a noise-resilient deep learning coherent photonic neural network layout that operates at 10GMAC/sec/axon compute rates and follows a noise-resilient training model. The coherent photonic neural network has been fabricated as a silicon photonic chip and its MNIST classification performance was experimentally evaluated to support accuracy values of >99% and >98% at 5 and 10GMAC/sec/axon, respectively, offering 6× higher on-chip compute rates and >7% accuracy improvement over state-of-the-art coherent implementations.

## Abstract (web summary)

The challenge of high-speed and high-accuracy coherent photonic neurons for deep learning applications lies to solve noise related issues. Here, Mourgias-Alexandris et al. address this problem by introducing a noise-resilient hardware architectural and a deep learning training platform.

## Introduction

The proliferation of Deep Learning (DL) workloads in today’s computational systems has triggered a new era in computing systems promoting the use of brain-inspired non-von-Neumann architectures, with the DL accelerators being currently considered as a key enabler for the efficient deployment of such workloads^1^. Among the researched DL accelerator technologies, neuromorphic integrated photonic circuits are constantly gaining interest due to their proven credentials to support time-of-flight latencies and THz bandwidths that may result to orders of magnitude higher computational and footprint efficiencies^2–4^. In this context, research efforts have mainly focused on the deployment and demonstration of the constituent building block technologies^5^ like weighting banks^6,7^ and activation functions^8–11^ as well as of complete linear neuron layouts^12–19^ that have so far largely relied on two broad architectural categories: (i) non-coherent setups, where typically one distinct wavelength is required per axon and optical power addition techniques are utilized for the summation functionality, leading to neuron layouts where the number of optical resource requirements scales linearly with its fan-in^20^, and (ii) coherent interferometric setups, where a single wavelength feeds the entire layout and the light carrier phase is employed for realizing signed weight values^16,21–23^.

The energy and area efficient promise of integrated photonic neural networks can, however, materialize only when significantly higher compute rates per axon are utilized compared to respective electronic Neural Network (NN) engines^2–4,12,13^. Recent analysis has indicated that the optimal Multiply-And-Accumulate (MAC)/sec/axon compute rates in optical setups should go well beyond the GHz regime^2–4^ in order to bring energy consumption down to the sub-pJ/MAC area. At the same time, high computational speeds have to be accommodated though on-chip elements for all constituent linear neuron functions in order to reap the benefits of low energy and low footprint integrated optics, including input vector generation, weighting and summation. Although the staggering computational power advances enabled by neuromorphic photonic circuitry, when using >10GMAC/sec/axon processing speeds, have been already witnessed through proof-of-concept demonstrations^15^, these have been realized through non-coherent architectures employing still non-integrated fiber-pigtailed building blocks for crucial NN functions. At the same time, their requirement for integrating and accurately controlling multiple resonant elements in high fan-in NN setups forms a significant hurdle towards their fully integrated version.

Fully on-chip optical neural networks have been so far only feasible through coherent architectural schemes, where, however, computational speeds are still far below the necessary targets and are struggling to enter the MHz regime^16,22,23^. On top of that, the accuracy values obtained by experimental coherent layouts reported so far have been limited to 90% and 76% for MNIST classification^22^ and vowel recognition^16^, respectively. Their rather limited performance in terms of both computational speed and accuracy is in direct relation to the overall noise that is inevitably present in any analog DL engine^24–31^, which is probably strengthened by the employed coherent architecture that comprises multiple cascaded Mach-Zehnder Interferometers (MZIs). Analog DL platforms have to cope by default with both deterministic and non-deterministic noise sources and the amount of noise increases significantly with operational speed. In the case of the experimental coherent-based layouts reported so far as neuromorphic platforms, their noise-resistive behavior is additionally counteracted by their circuit design that relies on Singular Value Decomposition (SVD) techniques applied over unitary optical layouts, which in turn comprise a mesh of multiple cascaded MZI following the Reck^32^ and Clements^33^ designs. In this way, the programming of multiple cascaded MZIs even for defining the weights of a single neuron is required, creating an increased sensitivity to fabrication errors that degrades the circuit fidelity and acts as an additional noise source.

In this work, we demonstrate experimentally noise-resilient deep learning at a record-high 10GMAC/sec/axon compute rate by utilizing a coherent silicon integrated circuit that combines a noise-tolerant linear neuron architectural scheme with noise-aware training methods. The silicon Coherent Photonic Neural Network (CPNN) circuit relies on the dual-IQ-modulator-based coherent linear neuron architecture recently proposed by us^21,34^, where a single on-chip weight value is simply defined by a phase shifting followed by an amplitude modulating element, significantly improving in this way its noise-tolerant characteristics compared to respective coherent layouts with cascaded MZIs. On-chip input vector data generation is realized by electro-optic travelling-wave Mach-Zehnder Modulators (MZM), with a single thermo-optic MZM and a single phase shifter providing the on-chip weighting per axon. Its performance has been validated within a DL layout that was trained with a noise-aware method^35,36^ for classifying hand-written images from the MNIST dataset, with its last two NN layers being implemented in the optical domain. Experimentally obtained accuracy values of >99% and >98% at 5 and 10GMAC/sec/axon compute rates, respectively, were obtained, even in the presence of highly noisy signals with a standard deviation of σ = 0.4. This validates the strong credentials of our integrated CPNN architectural scheme to combine its noise-tolerant design with noise-aware training models for leading to high-performance photonic DL layouts, outperforming state-of-the-art coherent-based demonstrations by 6 orders of magnitude with respect to on-chip compute rates per axon and by >7% with respect to obtained accuracy metrics.

## Results

### Concept and CPNN architecture

**Fig. 1:**
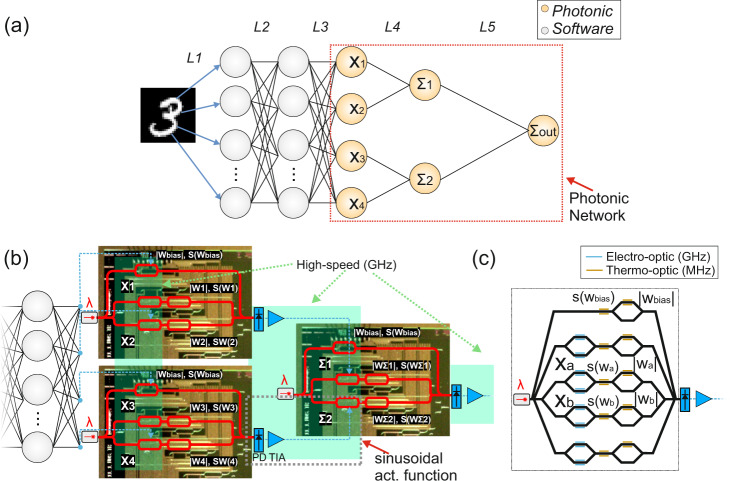
CPNN architecture and implementation. **a** Layout of the proposed CPNN, (**b**) a close-up view of its integrated photonic part based on the dual-IQ coherent linear neuron architecture, with the electro-photonic activation function module being highlighted with the red-dashed box and **c** the layout of the integrated 4-fan-in dual-IQ coherent linear neuron.

The layout of the CPNN designed for classifying MNIST images is illustrated in Fig. 1a. It comprises 2 cascaded ReLU convolutional layers (L1, L2) equipped with 32 and 64 3 × 3 kernels respectively, followed by a fully-connected ReLU feed-forward layer employed for data flattening (L3). A 4 × 2 photonic layer is employed as the L4, followed by a 2 × 1 photonic output layer denoted as L5. Both photonic layers utilize the *sin*^2^(*x*^2^) as activation function, so as to comply with respective optically implemented activations that rely on the use of a photodiode followed by a Mach-Zehnder Modulator (PD-MZM)^20^ at the output of an interferometric coherent architecture. A close-up view of the two photonic layers is depicted in Fig. 1b, where two identical silicon photonic chips are used for implementing the L4 and one additional chip is used for implementing the output layer L5. Each photonic neuron is identical and relies on the dual IQ architecture proposed in^21^, with its silicon-based deployment incorporating a 4-fan-in setup, as shown in Fig. 1c. A single CW laser source is split at the chip front-end to feed the bias branch as well as four axons. The CPNN design of Fig. 1a required the utilization of two silicon photonic axons at every chip for implementing the (*x*_1_, *x*_2_, Σ_1_) and (*x*_3_, *x*_4_, Σ_2_) layers within the L4 and the output layer L5. As shown in Fig. 1c, the input signals *x*_a_ and *x*_b_ of each axon are imprinted on the respective laser copies via an electro-optic MZM operating in the GHz regime. The weighting of each signal is performed individually via a thermo-optic phase shifter and a thermo-optic MZM that are responsible for the s(w_i_) sign and the weight absolute value |*w*_i_ | , respectively, with *i* = *a, b*. Despite the low bandwidth of few MHz that thermo-optic elements can achieve, during the inference their values are static, thus the computational rate of the CPNN is dictated only by the electro-optic MZMs operating in GHz. The main advantage of using thermo-optic elements is their lower insertion loss compared to the electro-optic elements, maintaining in this way the overall insertion loss at reasonable levels. The carrier of the realized dot product *x*_a_*w*_a_ + *x*_b_*w*_b_ interferes then with the bias signal before reaching the PD that has also a high bandwidth in the GHz regime, so that the dot product sign information imprinted on the phase of the summed signal can transform into an amplitude quantity where positive and negative values emerge as optical pulses above and optical dips below the bias signal, respectively^21^. The phase and the amplitude of the bias branch can be controlled by the s(w_bias_) and the |w_bias_ | , respectively. Finally, the electrical output of the PD is amplified through an electrical amplifier in order to drive the next layer, realizing at the same time the *sin*^2^(*x*^2^) activation function by exploiting the non-linearity of the MZM transfer function^20^, as shown in the inset of Fig. 1b. The non-injective behavior of the *sin*^2^(*x*^2^) is similar to the ReLU, which is also non-injective for x < 0, while the non-linear part has similarities with the sigmoid. The appropriate training framework for such activation functions has been published in^36^.

**Fig. 2:**
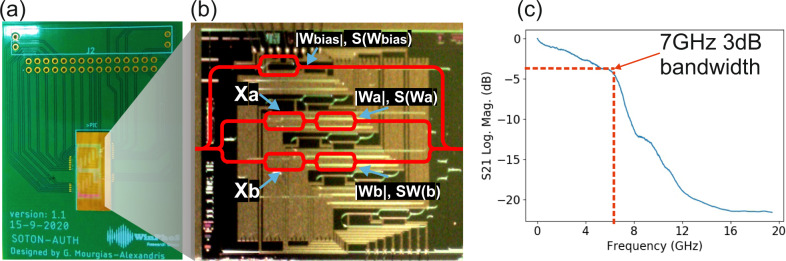
Silicon photonic neuron. **a** Photo of the packaged Si-Pho coherent neuron, **b** Microscope top-view photo of the SiPho chip, with the utilized part of the circuit highlighted in red and **c** Frequency response of the push-pull traveling-wave MZM, revealing a 3 dB bandwidth of 7 GHz.

Figure 2a illustrates a photo of the packaged Si-pho prototype, with the photonic chip mounted on an electrical Printed Circuit Board (PCB) that allows seamless electronic access to all the DC driven photonic components. A close-up microscope photo of the fabricated 4-fan-in Si-pho coherent neuron is depicted in Fig. 2b, highlighting with red lines the interferometric structures that were utilized simultaneously in the experimental implementation of the network shown in Fig. 1a (see S1). The electro-optic response of the travelling-wave MZMs used for the on-chip input vector data generation was characterized by measuring their frequency response, with an indicative measurement for one of the four MZMs depicted in Fig. 2c, revealing a 3 dB bandwidth of 7 GHz.

### MNIST classification at 10GMAC/sec/axon

**Fig. 3:**
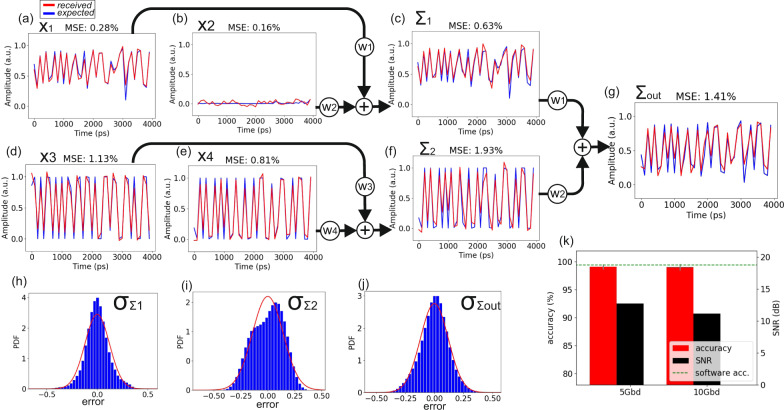
Experimental results for MNIST classification. **a**–**g** Time traces from the experimental evaluation of MNIST classification task on CPNN architecture illustrating the expected and the received signals, **h**–**j** noise distribution bar charts of each dot product Σi and the respective fitting with the Gaussian distribution and **k** accuracy and SNR measurements at 5 and 10GMAC/sec/axon.

Figure 3 depicts the experimental results obtained when the CPNN architecture was trained for MNIST classification tasks considering a noise-free hardware and signal environment, so as to validate the noise-related characteristics of the experimental CPNN platform through the comparative analysis between the expected and obtained waveforms. The time traces of Fig. 3a–g illustrate the signals originating from the software inferenced NN and their experimentally obtained counterparts with blue and red solid lines, respectively. More specifically, the time traces in Fig. 3a, b depict the *x*_1_ and *x*_2_ signals, while Fig. 3c illustrates the weighted sum of *x*_1_ and *x*_2_, denoted as Σ_1_. The Mean-Squared-Error (MSE) for *x*_1_, *x*_2_ and Σ_1_ signals was 0.28, 0.16 and 0.63%, respectively. Figure 3d–f illustrate the respective time traces for the *x*_3_, *x*_4_ and Σ_2_ signals of the 2nd neuron, with the corresponding MSE values being 1.13, 0.81 and 1.93%, respectively. The time trace of Σ_out_ signal that carries the sum of the weighted Σ_1_ and Σ_2_ values utilizing the weighting stage of the last NN layer is depicted in Fig. 3g, where the MSE was equal to 1.41%. Figure 3h, i and j depict the noise distribution of Σ_1_, Σ_2_, and Σ_out_, respectively, as well as the zero-mean best fit Gaussian distribution for each data batch, highlighted in red. Finally, the obtained classification accuracies and the calculated SNR values are depicted in Fig. 3k. The CPNN achieved an accuracy of 99.47% for the MNIST classification task when all its layers were implemented in a software environment, denoted by the dashed line as “software accuracy” in Fig. 3k. Its experimental validation with the last two layers implemented over the silicon photonic chip revealed an accuracy of 99.3% and 97.8% and a SNR of 14 and 12.4 dB at 5 and 10GMAC/sec/axon, respectively, confirming the low-noise characteristics of the proposed CPNN that allowed for only 0.17% and 1.67% degraded accuracy performance, respectively, compared to the software accuracy obtained within a noiseless environment. It is worth mentioning that the contribution of the software- and the hardware-implemented NN is analyzed in detail in supplementary note 2, where the whole NN has been implemented in software with noisy building blocks without any significant accuracy degradation.

### Noise-resilient CPNN

**Fig. 4:**
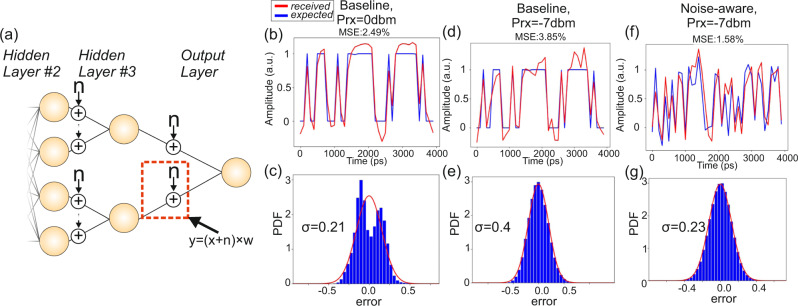
Experimental results for noise-aware training. **a** The modified photonic part of CPNN that incorporates AWGN sources on each *x*_i_ signal, **b**, **d**, **f** time trace and **c**, **e**, **g** noise distribution with the respective Gaussian fitting for : (i) the baseline model and 0 dBm at the Rx input, (ii) the baseline model and  −7dBm at the Rx (iii) noise-aware model −7dBm at the Rx.

Following the performance validation of the baseline CPNN model, the last two NN photonic layers were retrained following a noise-aware training model^35^ after introducing Additive White Gaussian Noise (AWGN). The AWGN had a mean value and a standard deviation that were set to be equal to the experimentally obtained noise characteristics of the silicon photonic circuitry. Figure 4a illustrates schematically how AWGN was inserted at every *x*_i_ in the CPNN layout in both photonic layers within the NN training model, in order to emulate the signal impairments originating by the photonic hardware platform. As can be seen, AWGN noise was considered to be added on every axon, so that a signal equal to *y* = (*x* + *n*) × *w* emerges from a single axon at the neuron output. The retraining procedure was implemented in the PyTorch software model of the CPNN, for a noise with a zero mean value and a standard deviation of σ = 0.4, revealing a software accuracy of 99.3% on the MNIST classification task.

Figure 4b–e illustrates the obtained experimental results of the baseline model when used with different experimental conditions and associated noise levels on the MNIST classification task, with Fig. 4f, g depicting the respective experimental results when the noise-aware training model was enforced. Figure 4b depicts the time trace of the CPNN output layer when the baseline model is applied, with the received optical power being equal to 0 dBm. The blue solid line represents the signal expected at the CPNN output when the network performs in software, while the red solid line shows the experimentally obtained pulse trace when an optical signal power of 0 dBm reaches the PD. The difference between the two waveforms had the distribution illustrated in Fig. 4c along with its Gaussian fitting, presenting a σ = 0.21 and a MSE = 2.49%. Figure 4d shows again the software-expected and experimentally obtained pulse traces when the optical signal gets attenuated by 7 dB prior reaching the photodiode, increasing in this way the noise of the photonic system that is primarily dominated by the Rx Trans-Impendence Amplifier (TIA) thermal noise contribution. In this case, the experimentally obtained waveform deviates even more from the expected software-based pulse trace, being the result of the higher system noise within a noise-agnostic baseline training. An increased MSE of 3.85% and a σ = 0.4 are obtained, with its distribution depicted in Fig. 4e. However, enforcing the noise-aware training model over the two photonic layers can significantly improve performance for the same noise level conditions, as can be verified by the time trace captured at the CPNN output layer and shown in Fig. 4f. The same −7 dBm optical power level was retained also in this case at the PD input so as to ensure identical noise levels with the respective results of Fig. 4d, e, with the distribution of the difference between the acquired and expected signals shown in Fig. 4g and validating a significantly improved performance over the baseline model, with σ and MSE values reducing to only 0.23 and 1.58%, respectively.

**Fig. 5:**
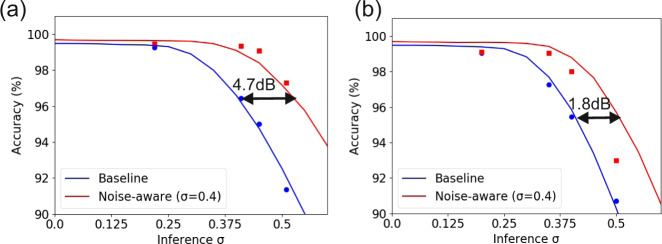
noise-aware training: simulation vs experiment. Accuracy on MNIST classification task versus noise standard deviation at **a** 5 and **b** 10GMAC/sec/axon. The solid lines represent the numerically simulated results and the points the experimentally acquired measurements.

A quantified comparison between the noise-aware MNIST classification model versus its baseline counterpart for different AWGN levels with a standard deviation ranging from σ = 0 up to σ = 0.6 was carried out both in software and experimental environment and the results of this analysis are illustrated in Fig. 5. The solid lines were derived from the software simulation model and the scatter points were derived from the experimentally validated DL platform, where increasing AWGN levels were obtained by attenuating the power level of the neuron output signal prior reaching the receiver. Figure 5a depict the achieved classification accuracies for the MNIST dataset at 5GMAC/sec/axon, with the blue and red points representing the experimentally obtained results from the baseline and noise-aware trained platform, respectively. As can be observed, the experimentally derived values follow closely the theoretically expected curves in both cases, validating the robustness of both the developed software framework and the effectiveness of the noise-aware model. The performance benefits of the noise-aware platform are revealed when the noise standard deviation exceeds the value of σ = 0.25, where the accuracy of the baseline model starts to degrade much faster reaching an accuracy of 95% at a σ = 0.4. At the same time, the accuracy of the noise-aware platform starts to degrade at significantly higher noise levels, remaining at >99% values even for noise standard deviations up to σ = 0.4. This implies that the performance advantages offered by the noise-aware platform can be either acquired as accuracy improvement over its baseline training model when the two schemes are evaluated for identical noise levels or as optical power savings when the same accuracy values are targeted by both schemes. More specifically, the 5GMAC/sec/axon noise-aware platform offers an accuracy improvement of 5.93% compared to the baseline model when the system noise has in both cases a standard deviation value of σ = 0.4. Alternatively, the noise-aware model can be considered as requiring a −11.7 dBm input optical power at the PD for ensuring the same 97.27% classification accuracy with the respective baseline scenario, where, however, a −7 dBm PD input optical power is needed. This highlights that the noise-aware platform can yield a power budget improvement of 4.7 dB that may translate into respective energy consumption benefits when a certain accuracy performance is targeted. Figure 5b depicts the same set of results for the case of a 10GMAC/sec/axon performing NN. Similar improvements are reported for noise values with a σ > 0.25, with the accuracy of the noise-aware platform remaining above 98% up to σ values of 0.4, revealing a best-case accuracy improvement of 2.54% or, alternatively, a power budget saving of 1.8 dB compared to the baseline scenario. It should be mentioned that the noise resiliency of the noise-aware method has been validated in detail in supplementary note 2, where the proposed training scheme enhances the noise resilience of the network using at the same time the least possible photonic hardware. This fact has been also verified on the CIFAR-10 dataset, where the usage of the noise-aware training allowed to approach the performance of traditional ReLU-based NN implementations.

## Discussion

**Fig. 6:**
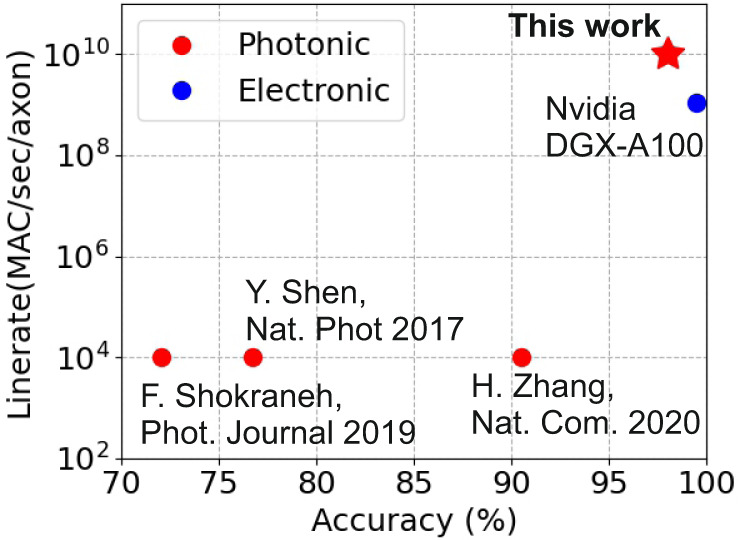
CPNN vs state-of-the-art. Reported performance of the state-of-the-art Nvidia DGX-A100 and few experimentally demonstrated coherent linear neuron engines in terms of line rate and classification accuracy.

The speed and accuracy performance benefits enabled by the proposed noise-resilient silicon photonic coherent DL platform can be clearly outlined when comparing with respective state-of-the-art coherent-based experimental layouts employed so far in neuromorphic applications. Figure 6 provides a pictorial representation of the combined MAC/sec/axon compute rate and accuracy metrics reported by coherent-based demonstrations so far^23,16^, and^22^, revealing that the experimental accuracy performance accomplished so far was only 72%, 76.7% and 90.5%, with the compute rate per axon never exceeding 10 kHz. All these coherent-based deployments relied on the use of cascaded MZI meshes following the SVD-based design over the unitary optical layouts proposed by Reck^32^ and Clements^33^. Our work is the first to follow an alternative on-chip coherent neuromorphic photonic architecture where a single column of weight values required by a single neuron can be enforced via a single respective column of optical components, avoiding in this way the use of cascaded photonic stages and safeguarding higher noise tolerance. Combined with a noise-aware DL training framework, the proposed silicon coherent neuromorphic platform allowed for the first time for 10GMAC/sec/axon on-chip compute rates and >98% accuracy values, outperforming all state-of-the-art coherent neurons by ~6 orders of magnitude in terms of per axon processing rates and by >7% in terms of accuracy performance. This brings its accuracy performance very close to the standards of state-of-the-art GPU platforms, as can be revealed in Fig. 6 by the classification performance metrics accomplished by a Nvidia DGX-A100 platform that executes the baseline MNIST classification model using the same NN architecture that was followed for the CPNN layout. Taking into account that the compute rate per axon in the latest Nvidia GPU is, however, one order of magnitude lower than the 10 GHz compute rate supported by the proposed silicon photonic neuromorphic platform (Fig. 6), the proposed CPNN equipped by noise-aware training models designates a promising framework for elevating DL performance metrics beyond state-of-the-art specifications of well-established DL technologies. This dual-IQ-modulator-based architecture, demonstrated here as a rather elementary silicon integrated chip that performs dot product operation between input and weight vectors, can also scale to input vector-weight matrix multiplication functions without sacrificing its noise-resilient properties^37^. This can pave the way towards a highly promising coherent neuromorphic photonic layout that may lead the race towards high-speed and high-accuracy chip-scale photonic DL engines, forming a promising alternative even to current well-established DL technology platforms. In doing so, a crossbar configuration seems to be the most promising candidate to realize up to 64-by-64 photonic vector by matrix multipliers with record-low loss and unitary fidelity (see S3).

We demonstrated experimentally a noise-resilient CPNN deployed as a silicon-integrated photonic chip and trained within a noise-aware feed-forward DL training framework, demonstrating the highest classification accuracy and the fastest compute rate per axon among all coherent linear neurons reported so far. The proposed photonic neuron architecture can be extended to support on-chip vector-matrix multiplication for implementing multi-neuron layers at chip-scale^37^ and can be also applied to alternative DL training models supporting Convolutional Neural Network (CNN) and Recurrent Neural Network (RNNs) configurations^38^. Moreover, its 10GMAC/sec/axon compute rate performance can be eventually increased by replacing the silicon MZM with a higher electro-optic bandwidth on-chip input vector data generation technology.

## Methods

### Design and fabrication of COLN

The COLN has been designed by using explicitly designed and tested photonic building blocks that are available at CORNERSTONE’s Process Design Kit (PDK). The chip has been fabricated in Cornerstone’s Silicon Photonic 220 nm platform and wire-bonded on a custom PCB. Each electro-optic MZM responsible for the generation of *x*_i_ signal relies on a push-pull asymmetric structure with 1.8 mm long phase shifters, while the heating elements are 560 um long. The insertion loss of the electro-optic MZMs is 8 dB, requiring 4.4 V on each arm to achieve a π-phase shift, while the thermo-optic MZMs has an insertion loss of 2 dB.

### Software-hardware interface

An in-house software tool was developed for interfacing the software and the hardware part of the proposed CPNN. Towards the software-hardware conversion, each signal was upsampled in order to achieve the required baud rate. Then, the signal was pre-equalized by means of 11-tap Feed-Forward Equalizer (FFE) and quantized with an 8-bit format before reaching a M8195a AWG from Keysight with 65GSa/s sampling rate, 8-bit precision and 25 GHz 3 dB bandwidth. The output of the PD is sampled by means of an DSOZ634a real-time oscilloscope with 33 GHz 3 dB bandwidth, 80GSa/s sampling rate and 10-bit resolution. Afterwards, the hardware-software interface performs time recovery to the captured waveform and then a Gaussian-shaped filter is applied. The filtered signal is downsampled to 1 sample before entering the software-based NN.

### Noise-aware training model

Τhe CPNN was implemented in software by means of PyTorch framework. All models were initialized using the Xavier initialization with a gain of 2 and the weights were optimized for 20 epochs using a variant of stochastic gradient descent, i.e. the Adam optimizer. Finally, the size of each batch was equal to 256 and the learning rate was set to 0.0001. For the training of noise-aware model, the experimentally measured standard deviation and the distribution of system’s noise was used to emulate the experimental conditions. The experimental measurements of noise were performed within a certain range of received optical power, resulting in different σ values ranging in [0, 0.7]. Then, the CPNN was trained by introducing noise on each axon based on the experimental findings, resulting in three different models trained with σ = 0.2, 0.4 and 0.7. The simulation and the experimental validation of three models shown that the model with σ = 0.4 has the best performance across the range of σ = [0, 1], establishing this model towards the experimental validation of noise-resilient capabilities of the CPNN. Note that the noise during the training procedure was generated by using the randn() function of PyTorch.

## Supplementary information

Supplementary information

## Data Availability

The data that support the findings of this study are available from the corresponding authors on reasonable request.

## Code availability

The code that used for training the ML models is available on GitHub: https://github.com/georgemourgias/noise_aware_cpnn.
