# Supplementary material for: Noise-resilient and high-speed deep learning with coherent silicon photonics
Source: Nat Commun. 2022 Sep 23;13:5572. doi: 10.1038/s41467-022-33259-z (PMC9508134; doi:10.1038/s41467-022-33259-z)
Supplement: Supplementary file 1 — Supplementary information [file 41467_2022_33259_MOESM1_ESM.docx]

Supplementary information for "Noise-resilient and high-speed deep learning with coherent silicon photonics"

G. Mourgias-Alexandris*^1,2^, M. Moralis-Pegios^1,2^, A. Tsakyridis^1,2^, S. Simos^1,2^, G. Dabos^1,2^, A. Totovic^1,2^, N. Passalis^1^, M. Kirtas^1^, T. Rutirawut^3^, F. Y. Gardes^3^, A. Tefas^1^ and N. Pleros^1,2^

*^1^Department of Informatics, Aristotle University of Thessaloniki, 54124, Thessaloniki, Greece*

*^2^Center for Interdisciplinary Research and Innovation, Aristotle University of Thessaloniki, Greece*

*^3^Optoelectronics Research Centre, University of Southampton, Southampton, SO17 1BJ, UK*

[*mourgias@csd.auth.gr](mailto:*mourgias@csd.auth.gr)

# **S1. Experimental setup used for the validation of the CPNN**

Supplementary Figure 1 depicts the experimental setup used for the experimental deployment of the MNIST classification task on three individual Coherent Photonic Neural Networks (CPNNs). In this coherent photonic layout that follows the architectural principles described in [1], the light entering the photonic neuron through a grating coupler gets split in a 50/50 splitter before being forwarded in the bias branch and the *x*_i_*w*_i_ stage. The bias branch is implemented through a Thermo-Optic Mach-Zehnder Interferometer (TO-MZI) with 600um long arms followed by a 550um long TO Phase Shifter (TO-PS), while the *x*_i_*w*_i_ stage comprises 4 identical branches. Each branch implements the *x*_i_ data imprinting through an electrooptic (EO) push-pull asymmetric Mach-Zehnder Modulator (MZM), where each EO phase shifter is 1900um long and is operated in travelling wave configuration, featuring 7GHz 3dB bandwidth and average insertion losses of 8dB. The *w*_i_ weight imprinting is realized through a TO-MZI and the *w*_i_ sign imprinting through a TO-PS, while each device features dimensions equal to the similar ones on the bias branch. The incoming light beam to the *x*_i_*w*_i_ stage gets split into 4 identical copies, through two cascaded 50/50 splitters, and after data and weight imprinting is again recombined into a single light beam through two cascaded 50/50 combiners. Finally, light originating from the bias and the *x*_i_*w*_i_ stage gets recombined in a 50/50 combiner. It should be noted that the sign of the weight is imprinted through the control of the phase of the light beams, with negative values realized by inducing a Δφ=π in relation with the bias branch, and positive values by matching the bias signal phase. The constructive or destructive interference of the signals originating from the *x*_i_*w*_i_ stage with the bias branch allows translation of the weight sign information into the final output.

For the on-chip implementation of the weighted summation of *x*_a_, *x*_b_ for each one of the 3 respective CPNNs the following experimental procedure was followed. A light beam at λ_1_=1554.55 nm was injected to the SiPho chip via a TE grating coupler. The |*w*_bias_| TO-MZI was used to control the bias branch amplitude, while the PS_bias_ was used to control its phase. Two EO-MZMs were used to optically imprint the corresponding *x*_a_ and *x*_b_ values originating from the NN, while the respective weighting values were imprinted by the TO MZIs |*w*_a_| and |*w*_b_| and their corresponding signs through controlling the TO-PS PS_a_ and PS_b_, respectively. In order to interface the *x*_a_ and *x*_b_ data originating from the NN to the integrated 4-fan-in COLN, their respective waveforms were upsampled from 1 to 60, 12 or 6 samples per symbol (sps), corresponding to operational data-rates of 1,5 and 10Gbaud, and were then filtered by a Gaussian filter. The resulting signals were finally quantized with 8-bit resolution before being uploaded to Keysight's M8195a Arbitrary Waveform Generator (AWG) operating at 60GSa/s. The 2 output signals and their differential copies originating from the AWG, were then forwarded to 4 SHF100BO-ML RF amplifiers to drive simultaneously the 2 push-pull MZMs with approximately 3Vpp. The SiPho output optical signal was converted to the electrical domain by the means of a PIN photodetector with 54GHz 3dB bandwidth, 45A/W responsivity and 25nA dark current when is biased at 4V.

The electrical signal was captured by a Keysight DSAZ634a Real Time Oscilloscope (RTO) with 80GSa/s and 33GHz bandwidth. The received signal was time-synchronized with the expected signal, and was then filtered with a Gaussian filter before being downsampled to 1sps and forwarded to the next NN layer. Each accuracy measurement for the CPNN and the noise-aware model was realized by capturing 7,476,480 samples by means of an RTO. The experimental validation of CPNN architecture on MNIST classification task was performed by launching 0dBm optical power to the photodiode, while the reported standard deviation values in the noise-aware model evaluation were achieved between -14 and 0dBm received optical power.


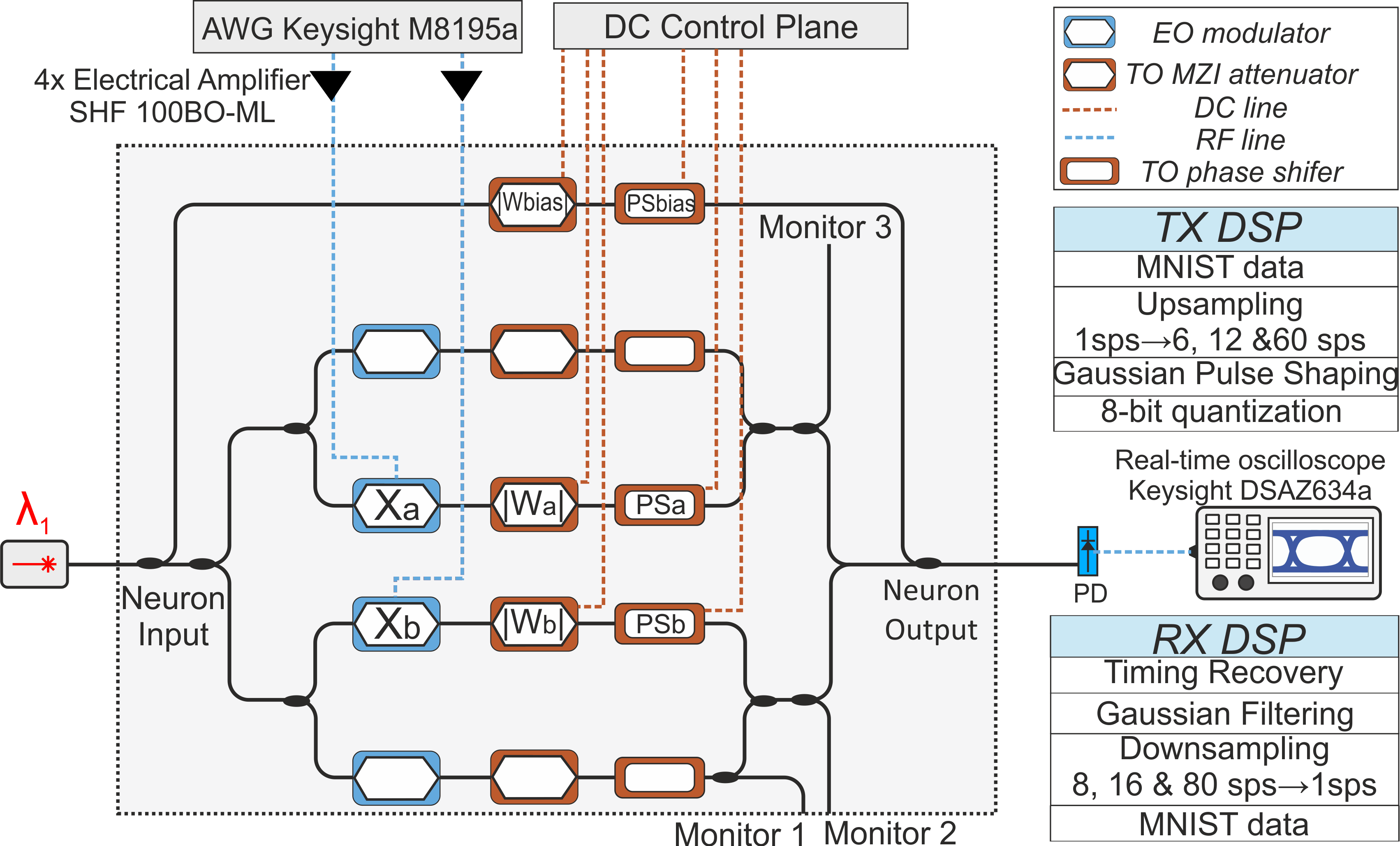


Supplementary Figure 1: **CPNN experimental setup.** Experimental setup used for the evaluation of the CPNN, including the signal processing interfaces required to interface the CPNN photonic chip with the digital world

# **S2. CPNN classification impact and noise-aware training**

Within this section, the impact of implementing NNs on the CPNN and of the proposed noise-aware training scheme on the final classification accuracy of a MNIST NN-based classifier, was assessed by conducting a series of additional experiments. Moreover, the performance of an NN implemented exclusively with software simulated CPNNs on more sophisticated tasks like CIFAR‑10 was evaluated.

Starting with the contribution of the integrated CPNN on the MNIST classification task, we conducted a series of experiments to quantify the contribution of each of the constituent NN layers on the final classification accuracy. First, the first two convolutional layers implemented in software (L1+ L2 in Sup. Fig. 2) were completely removed to evaluate their contribution to the final
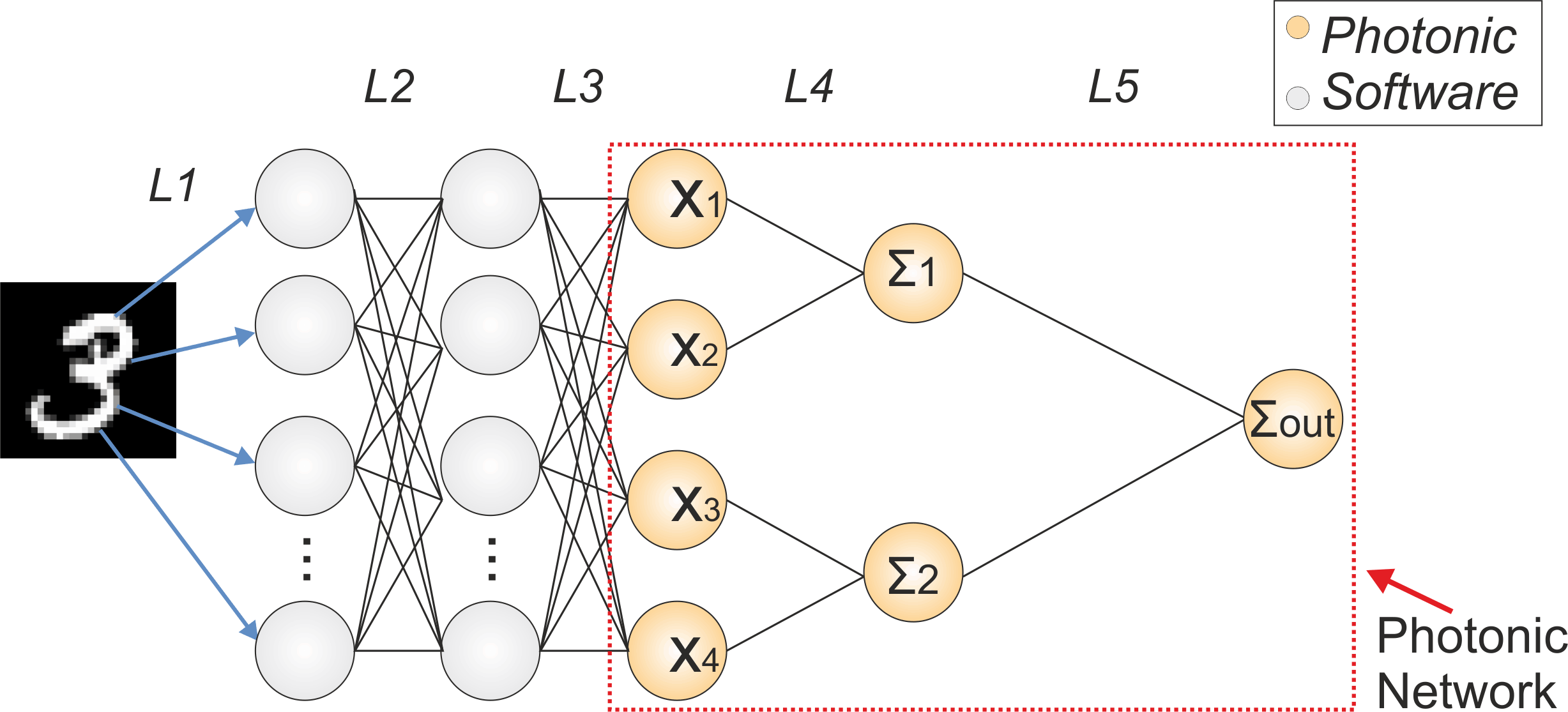


Supplementary Figure 2: **NN layout of CPNN.** CPNN architecture including 2 convolutional layers (L1, L2) and 3 feed-forward layers (L3, L4 and L5)

classification accuracy for the case of the 10Gbaud photonic NNs, where a classification accuracy of 97.8% is reported (Fig. 3k in the manuscript). This setup was followed instead of keeping only the software-based layers because it is not possible to measure any meaningful classification metrics without its final output layer, that is responsible for making the classification decision. As shown in Supplementary Table 1 below, the accuracy of the network without the software-implemented convolutional layers dropped by about 3%.

Supplementary Table 1 : **Contribution of convolution layers.** Accuracy comparison of CPNN, with and without CNN layers

| **Model** | **Accuracy** |
| --- | --- |
| Convolutional + Fully Connected (L1-L5) | 97.89 |
| Fully Connected only (L3-L5) | 94.90 |

Followingly, in order to quantify : (i) the performance contribution of each NN layer (ii) the degradation originating from the noise-sources and non-linear behavior of the analog photonic accelerator, we developed a Pytorch based simulation framework, that models the limited frequency response and the major noise sources of the photonic link and extensive analyzed through simulation experiments both the impact of the photonic-simulated layers and the accuracy contribution of each of the constituent NN layers. This was accomplished by gradually removing NN layers, from the MNIST classifier network, in order to evaluate the impact of each layer in the final classification accuracy, as well as quantify the contribution of the proposed noise-aware training methods. Three different setups were evaluated:

1. A network that is implemented using the aforementioned photonic simulation framework (abbreviated as *“Baseline”*) and without enforcing any noise-aware training scheme
2. The same network using the proposed noise-aware training approach (abbreviated as *“Proposed”*)
3. A network implemented without the use of the simulated photonic components (abbreviated as *“Software”*), i.e., as it would be implemented in a modern GPU

For all these experiments, we used a slightly different architecture that is more balanced between layers, while also having an additional FC layer (i.e., L3-L5 has been replaced with L3-L6 to avoid large discrepancies between successive layers that can act as information bottleneck), in order to allow us to better evaluate the impact of the depth of the network. The number of convolutional filters/neurons of the first two convolutional (L1, L2) and fully connected layers (L3, L4) are set to 64, followed by a fully-connected layer with 32 neurons (L5) and a final decision neuron (L6). The results are provided in Supplementary Table 2 below:

Supplementary Table 2 :**Contribution of CPNN layers.** Comparison of achieved MNIST accuracies, for (i) various NN depths (ii) Simulated photonic (Baseline), Simulated photonic with Noise-aware training (Proposed), Conventional GPU Software implementation (Software)

| **Model** | **Baseline** | **Proposed** | **Software** |
| --- | --- | --- | --- |
| All Layers (L1-L6) | 93.53 | **99.68** | 99.47 |
| Without Convolutional Layers  (L3-L6) | 98.68 | **99.47** | 98.73 |
| Without Convolutional and One Fully Connected  *(*L4-L6) | 97.63 | **98.84** | 98.68 |
| Without Convolutional and Two Fully Connected  *(*L5-L6) | 88.64 | **95.32** | 95.53 |

The proposed method in all cases significantly improves the photonic accuracy, almost matching in many cases the accuracy of a software network. Furthermore, the results also highlight the difficulty in training photonic networks with larger convolutional layers. Various phenomena such as vanishing gradients or excessive noise can lead to sub-optimal training. Indeed, using convolutional layers in the baseline architecture leads to lower accuracy, i.e.,93.53% accuracy instead of 98.68%, despite having a more powerful architecture that can perform better (this is demonstrated in the software implemented network). This was the primary motivation behind designing and implementing the proposed method. When the proposed method is used, the network's performance is restored and provides the network's total capacity, similar to the software case. As a result, implementing the entire MNIST network in the optical domain and applying a baseline training method indeed brings a non-negligible accuracy degradation of ~6% compared to the software-based implementation. On top of that, employing the noise-aware training technique over the all-optical NN can compensate for this accuracy degradation. This is valid even if the convolutional and the two fully-connected layers are removed so that only the final NN layer is retained, a scenario that closely follows the use of the photonic NN chip in the last two layers of the network utilized in our experiments. In this case, the baseline scenario experiences a drop of about 5% compared to where all layers are incorporated, with a respective ~4% drop also experienced by the software-based implementation. However, the employment of the noise-aware scheme allows the photonic NN to recover from its "baseline" performance close to the "software-based" performance. This also indicates that just the use of a last photonic fully-connected layer within the NN without incorporating any additional convolutional and fully-connected layers at the NN front-end, is responsible for an accuracy of 95% that can also be experimentally met by the photonic NN when the proposed noise-aware training scheme at the rate of 10Gbaud is employed.

Before delving deeper into the noise-aware training methodology, it is necessary to further elaborate on the various noise sources stemming from the high-bandwidth electrophotonic hardware. In particular, the noise profile of each noise source used in the noise-aware modeling has been formulated by considering the following noise sources:

1. *Relative Intensity Noise (RIN)*: This noise stems from the laser source that used to feed with light each neuron. The laser itself introduces arbitrary power fluctuations over time [1], resulting in RIN.
2. *Quantization noise*: The photonic neuron is interfaced with the electronic equipment through DACs and ADCs, that are responsible for converting the digital signal to analogue and vice versa. These conversions, however, come at the cost of quantization noise [2], where the limited precision of each converter results in information loss.
3. *Shot + Johnson noise*: The output of the silicon photonic neuron is coupled into a photodetector to realize the required optoelectronic conversion. The physical properties of the photodetector introduces mainly 2 different types of noise into the system, known as shot and Johnson noise [3], respectively.
4. *Thermal noise*: Thermal noise is present on any electronic circuit and it’s proportional to the temperature of the device. Usually, the thermal noise dominates such systems, while its approximation fits perfectly to the Additive White Gaussian Noise (AWGN).

Without loss of generality, all the above-mentioned noise sources can be approximated as AWGN accumulated from photonic neuron’s constituent photonic devices. Indeed, in [4] the validity of using AWGN models to emulate the noise profile of neuromorphic photonic hardware has been successfully demonstrated. Finally, this modelling method is perfectly aligned with a similar one that was employed in optical interconnect systems [5]. Regarding the employed noise-resilient training approach, it is worth mentioning that is incorporated into the regular neural network training process by introducing non-differentiable noise during the feed-forward process. Therefore, during the feed-forward pass, the signal quality of each input is impaired by the introduced noise, allowing the adaption of the neural network's weights in the training process to compensate for the noise that has been added. It is worth noting that the proposed method does not modify the backpropagation, allowing its implementation with any standard state-of-the-art DL framework. For all the training and simulation experiments conducted in this paper, the PyTorch library was used. The noise was modelled using the torch.randn() function, while the introduced corruption was appropriately scaled according to the signal statistics.

The Adam optimizer was used for the experiments using the MNIST dataset, adopting the learning rate scheduling technique (10 epochs with a learning rate of 10^-3^, 100 epochs with a learning rate of 10^-4^ and additional 100 epochs with a learning rate of 10^-5^), and a batch size of 256. For the CIFAR dataset, the procedure was similar but with limited epochs to 5, 10 and 20 respectively, using a batch size of 64 that results in a similar number of network updates as for the MNIST dataset. Using a smaller batch size allows for accelerating the convergence of the network since more updates are performed for each epoch.

The contribution of the noise-aware training across different noise intensities was evaluated with various experiments where the whole, experimentally validated, network is implemented with simulated photonic components. In particular, two different setups were realized:

a) A network that is implemented using the aforementioned photonic simulation framework (abbreviated as “Baseline”) and without enforcing any noise-aware training scheme.

b) The same network using the proposed noise-aware training approach (abbreviated as "Proposed").

The acquired classification accuracy results for different noise levels are summarized Supplementary Table 3.

Supplementary Table 3 : **Noise- aware performance.** Performance comparison of NN trained with (Proposed) and without (Baseline) the proposed noise-aware training scheme

| **Noise Level (σ)** | **Baseline** | **Proposed** |
| --- | --- | --- |
| 0 | 99.42 | **99.47** |
| 0.1 | 99.42 | **99.52** |
| 0.2 | 99.36 | **99.42** |
| 0.3 | 96.47 | **99.15** |
| 0.4 | 89.80 | **96.31** |
| 0.5 | 83.75 | **93.37** |

In all cases, the proposed noise-aware training method leads to significant improvements when applied on top of the baseline network. The experimentally measured noise corresponds to the underlined case (σ=0.4). Additional experiments were also conducted with a larger architecture to evaluate the impact of network depth on the accuracy of the network. The number of convolutional filters/neurons of the first two convolutional and fully connected layers are set to 64, followed by a fully-connected layer with 32 neurons and a final decision neuron. The results are provided in the Supplementary Table 4 below:

Supplementary Table 4: **NN depth contribution.** Performance comparison of more complicated and wider NN trained with (Proposed) and without (Baseline) the proposed noise-aware training scheme

| **Noise Level (σ)** | **Baseline** | **Proposed** |
| --- | --- | --- |
| 0 | 99.47 | **99.57** |
| 0.1 | 99.36 | **99.63** |
| 0.2 | 99.26 | **99.63** |
| 0.3 | 97.58 | **99.57** |
| 0.4 | 93.63 | **99.68** |
| 0.5 | 86.27 | **99.57** |

These results indicate that the use of larger network architectures increases the noise resilience of the network, even when the proposed noise-aware training is not used. As such, the noise sensitivity increases as the network architecture becomes shallower, making this scheme even more important for all-optical NN implementations where the photonic NN technology is still not mature enough to support fan-ins for the deployment of DNNs.

The classification capabilities of the CPNN hardware have also been tested on CIFAR-10 dataset. All experiments were conducted by simulating the photonic hardware that implements two convolutional layers with 64 filters, followed by two fully connected layers with 64 and one with 32 neurons and a final decision neuron. The simulations do not use any traditional software-based layers for the optical networks, i.e., all layers are implemented in the optical domain using the aforementioned simulation framework. The results are summarized in Supplementary Table 5.

Supplementary Table 5 : **CIFAR10 classification.** Classification accuracy results for the CIFAR-10 dataset for: Simulated photonic (Baseline), Simulated photonic with Noise-aware training (Proposed), Conventional GPU Software implementation (Software)

| **Model** | **Accuracy** |
| --- | --- |
| Baseline | 80.55 |
| Proposed | **83.50** |
| Software | 83.90 |

The proposed noise-resilient method significantly improves the networks' accuracy by increasing their resilience to noise, despite solving a significantly more complex problem. On top of that, the proposed method almost matches the accuracy of a traditional ReLU-based network implemented in software, highlighting once again the effectiveness of the proposed method.

# **S3. CPNN scalability & energy requirements**

The demonstrated CPNN allows for the encoding of a neuron with 4 inputs and a single output. However, Deep Neural Networks are built on multiple layers with multiple neurons each, requiring accelerators able to carry out multiplication operations of matrices with much higher dimensions. To this end, we envision the spatial expansion of the proposed CPNN into both the vertical and horizontal plane, towards a coherent photonic *N*-by-*M* crossbar architecture, capable of supporting *M* neurons with *N* inputs, while following the layout depicted in Sup. Fig. 3 (a). The envisioned crossbar will rely on the same architectural principles as the demonstrated 4-input CPNN, offering significant advantages over the state-of-the-art coherent architectures [6], [7], [8], [9], such as: 1) the direct and optimal fidelity mapping of the NN parameters onto the photonic platform, 2) low complexity and linear loss dependence to the employed active building blocks responsible for the NN parameters imprinting. The latter will lead to tremendous insertion loss savings, allowing the implementation of a highly scalable coherent layout with extraordinary energy and computational density performance.


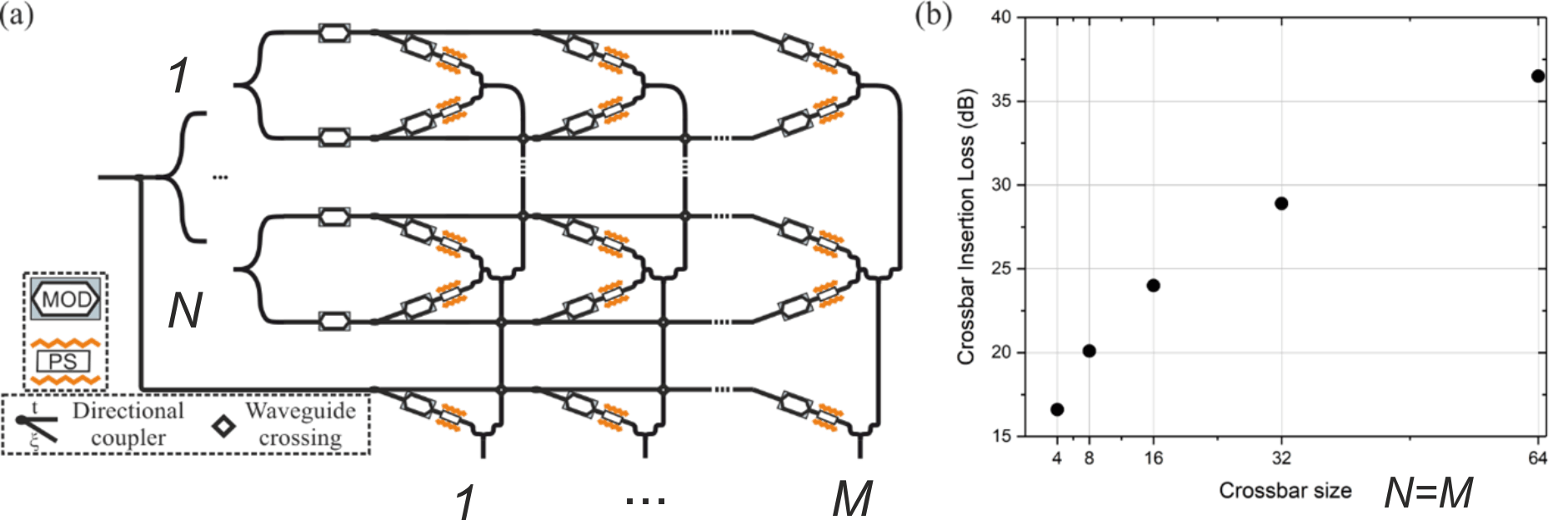


Supplementary Figure 3: **Photonic Crossbar.** (a) the envisaged N-by-M coherent crossbar array and (b) its insertion loss as a function of fan-in

To provide quantitative information about the scalability credentials of our technology when extended into a crossbar layout, we evaluate the insertion loss performance of a photonic *N-by-N* crossbar configuration that utilizes the electro-optic input MZM and thermo-optic weighting technology employed in our photonic circuit. More specifically, for our analysis we considered the experimentally derived excess losses for the input and weight imprinting devices i.e., 8 dB per electro-optic MZM, 2 dB per thermo-optic MZM, and 0.06 dB, 0.1 dB and 0.02 dB per MMI coupler [10], directional coupler [11] and waveguide crossing [12], respectively. Figure S3(b) illustrates the total insertion loss of square crossbar layouts, where N=M, for crossbar implementations ranging from N=2 to N=64. It can be clearly identified, that our neuromorphic architecture can scale to a matrix-vector-multiplication engine for matrix dimensions of 32x32, with reasonable insertion losses of 28.9 dB, as such supporting a total number of 1024 multiplications that yield a total MAC rate of 10.2 TMAC/sec when considering the speed of 10GMAC.

Both the 4-input CPNN and the envisaged coherent crossbar necessitate Digital-to-Analog-Converters (DACs) and Analog-to-Digital Converters (ADCs) to realize the required ElectroOptic (EO) and OptoElectronic (OE) conversions, that will effectively bridge the electronic circuitry with the photonic accelerator. Moreover, in order to drive the MZM modulators an RF amplifier after the DAC is required, along with a Transimpedance Amplifier (TIA), following the PD and preceding the ADC. Table S6, summarizes the energy efficiency of the constituent electronic building blocks, considering a processing rate of 10 GMAC/s, concluding also to general expressions for the achieved energy efficiencies of the envisioned N-by-1 linear neuron and N‑by‑M crossbar layouts. It is worth mentioning that the weight imprinting elements are not included in this analysis since their static functionality can be easily realized through zero-power consuming phase change materials [13].

Supplementary Table 6 :**System’s energy efficiency:** Breakdown and total energy efficiency of N-by-1 neuron and N-by-M Xbar architecture, including E/O/E interfaces. All values are in pJ/MAC.

| Device | *N*-by-*1 (Linear Neuron)* | | | *N*-by-*M (Xbar)* | |
| --- | --- | --- | --- | --- | --- |
| DAC | *N* × 0.5 [14] | *N* × 0.5  [14] | | | |
| RF Amp | *N* × 19 [15] | *N* × 19 [15] | | | |
| TIA | 0.24/ *N* [16] | *M* × 0.24 [16] | | | |
| ADC | 0.21 / *N* [17] | | *M* × 0.21 [17] | | |
| Total overhead | (*N* ×1.95 + 0.43 / *N*) pJ/MAC | | | | (*N* ×1.95 + *M* × 0.43) pJ/MAC |

**Supplementary References**

1. I. Joindot, “Measurements of relative intensity noise (RIN) in semiconductor lasers,” Journal de Physique III, vol. 2, no. 9, pp. 1591–1603, 1992.
2. S. Varughese et al., “Frequency dependent enob requirements for 400g/600g/800g optical links,” Journal of Lightwave Technology, 2020.
3. N. C. Harris et al., “Noise characterization of a waveguide-coupled MSM photodetector exceeding unity quantum efficiency,” Journal of lightwave technology, vol. 31, no. 1, pp. 23–27, 2012.
4. N. Passalis, M. Kirtas, G. Mourgias-Alexandris, G. Dabos, N. Pleros, and A. Tefas, “Training noise-resilient recurrent photonic networks for financial time series analysis,” Eur. Signal Process. Conf., vol. 2021-January pp. 1556–1560, 2021.
5. R. -J. Essiambre, G. Kramer, P. J. Winzer, G. J. Foschini and B. Goebel, "Capacity Limits of Optical Fiber Networks," in Journal of Lightwave Technology, vol. 28, no. 4, pp. 662-701, Feb.15, 2010, doi: 10.1109/JLT.2009.2039464.
6. G. Mourgias-Alexandris et al., "Neuromorphic Photonics With Coherent Linear Neurons Using Dual-IQ Modulation Cells," in Journal of Lightwave Technology, vol. 38, no. 4, pp. 811-819, 15 Feb.15, 2020.
7. G. Giamougiannis et. al., "Coherent photonic crossbar as a universal linear operator", submitted to Laser and Photonics Reviews (2021)
8. N. Pleros et.al.," Compute with Light: Architectures, Technologies and Training Models for Neuromorphic Photonic Circuits", ECOC 2021
9. M. Moralis-Pegios, G. Mourgias-Alexandris, A. Tsakyridis, G. Giamougiannis, A. Totovic, G. Dabos, N. Pleros, "Coherent photonic neuromorphic computing for high-speed deep learning applications," Proc. SPIE 12007 Op-tical Interconnects XXII, 1200706 (5 March 2022). doi.org/10.1117/12.2606041
10. Z. Sheng, Z. Wang, C. Qiu, L. Li, A. Pang, A. Wu, X. Wang, S. Zou, F. Gan "A Compact and Low-Loss MMI Coupler Fabricated With CMOS Technology," IEEE Photonics Journal, 4 (6), 2272-2277 (2012) doi: 10.1109/JPHOT.2012.2230320.
11. B. Sharma, K. Kishor, A. Pal, S. Sharma and R. Makkar, "Design and simulation of ultra-low loss triple tapered asymmetric directional coupler at 1330nm," Microelectronics Journal 107, 104957 (2021). <https://doi.org/10.1016/j.mejo.2020.104957>.
12. Y. Ma, Y. Zhang, S. Yang, A. Novack, R. Ding, A. Eu-Jin Lim, Guo-Qiang Lo, T. Baehr-Jones, and M. Hochberg, "Ultralow loss single layer submicron silicon waveguide crossing for SOI optical interconnect," Opt. Express 21 (24), 29374-29382 (2013). <https://doi.org/10.1364/OE.21.029374>.
13. J. Feldmann, M. Youngblood, M. Karpov, H. Gehring, X. Li, M. Stappers, M. Le Gallo, X. Fu, A. Lukashchuk, A.S. Raja, J. Liu, C.D. Wright, A. Sebastian, T.J. Kippenberg, W.H.P. Perince and H. Bhaskaran, “Parallel convolutional processing using an integrated photonic tensor core”. Nature 589, 52–58 (2021).
14. P. Caragiulo, O. E. Mattia, A. Arbabian and B. Murmann, "A Compact 14 GS/s 8-Bit Switched-Capacitor DAC in 16 nm FinFET CMOS," 2020 IEEE Symposium on VLSI Circuits, 2020, pp. 1-2, doi: 10.1109/VLSICircuits18222.2020.9162776.
15. S. Nakano et al., "A 180-mW Linear MZM Driver in CMOS for Single-Carrier 400-Gb/s Coherent Optical Transmitter," 2017 European Conference on Optical Communication (ECOC), 2017, pp. 1-3, doi: 10.1109/ECOC.2017.8346186.
16. C. Schow, A. Rylyakov, C. Baks, F. Doany, and J. Kash, “25-Gb/s 6.5-pJ/bit 90-nm CMOS-Driven Multimode Optical Link,” IEEE Photonics Technol. Lett. 24(10), 824–826 (2012).
17. E. Swindlehurst et al., "An 8-bit 10-GHz 21-mW Time-Interleaved SAR ADC With Grouped DAC Capacitors and Dual-Path Bootstrapped Switch," in IEEE Solid-State Circuits Letters, vol. 2, no. 9, pp. 83-86, Sept. 2019, doi: 10.1109/LSSC.2019.2931440.
